# A trainable clustering algorithm based on shortest paths from density peaks

**DOI:** 10.1126/sciadv.aax3770

**Published:** 2019-10-30

**Authors:** Diego Ulisse Pizzagalli, Santiago Fernandez Gonzalez, Rolf Krause

**Affiliations:** 1 Institute for Research in Biomedicine, Faculty of Biomedical Sciences, Università della Svizzera italiana, CH6500 Bellinzona, Switzerland.; 2 Institute of Computational Science, Università della Svizzera italiana, CH6900 Lugano, Switzerland.

## Abstract

Clustering is a technique to analyze empirical data, with a major application for biomedical research. Essentially, clustering finds groups of related points in a dataset. However, results depend on both metrics for point-to-point similarity and rules for point-to-group association. Non-appropriate metrics and rules can lead to artifacts, especially in case of multiple groups with heterogeneous structure. In this work, we propose a clustering algorithm that evaluates the properties of paths between points (rather than point-to-point similarity) and solves a global optimization problem, finding solutions not obtainable by methods relying on local choices. Moreover, our algorithm is trainable. Hence, it can be adapted and adopted for specific datasets and applications by providing examples of valid and invalid paths to train a path classifier. We demonstrate its applicability to identify heterogeneous groups in challenging synthetic datasets, segment highly nonconvex immune cells in confocal microscopy images, and classify arrhythmic heartbeats in electrocardiographic signals.

## Teaser

By considering knowledge as a path, machines and humans can collaboratively analyze complex biomedical datasets.

## INTRODUCTION

Clustering algorithms aim at automatically grouping points. This task is critical for extracting knowledge from complex datasets of unlabeled data, which are commonly produced by empirical studies (*1*) and are impossible to analyze manually (*2*). The identified groups can indicate the presence of multiple phenomena (i.e., populations) in the dataset. Moreover, the structure of each group (hereafter, shape) can indicate the relationship between the included points. Because clustering is a problem without a unique solution, results depend on different metrics for point-to-point similarity and rules for point-to-cluster association. However, metrics and rules generally restrict the set of admissible solutions. As a consequence, when metrics and rules are not chosen appropriately, clustering artifacts are introduced. This is especially relevant when analyzing datasets that have points from populations with arbitrary and heterogeneous structure.

For example, common implementations of the broadly used *K*-means algorithm (*3*) use the Euclidean distance as a metric for point-to-point similarity and point-to-cluster association. Despite the efficiency of *K*-means in clustering linearly separable groups (i.e., globular-like), artifacts can be introduced when attempting to separate nonconvex and nested shapes.

Methods based on density overcome these limitations. Arbitrary shapes are reconstructed according to a density-connectivity criterion. For example, DBSCAN (density-based spatial clustering of applications with noise) (*4*) considers two points belonging to the same cluster if a sufficient number of points in a neighborhood are common (density reachable). As a result, the association rule of DBSCAN correctly identifies clusters with any shape having sufficient density. However, when two or more clusters are present and in close proximity, a wide density reachability threshold may join them, while an excessively strict threshold may fail in detecting the clusters. Generally, algorithms like DBSCAN depend on a density-connectivity threshold, which can be difficult to adapt for specific dataset and applications.

Recently, Rodriguez and Laio (*5*) proposed a remarkable strategy that achieves clustering by finding density peaks (CDP). CDP addresses the limitations of DBSCAN by initially finding density peaks and using them to separate clusters. Density peaks are considered as points surrounded by enough other points with lower density. Hence, the distance between two density peaks should be higher than the distance between another arbitrary point and its closer point with a higher density. On the basis of this intuition, CDP identifies density peaks by detecting the outliers of a density-distance plot. This task can be performed either automatically or manually by inspecting the density-distance plot. Because of the simplicity of this rule, CDP can be applied whenever density can be measured, which holds in a wide range of applications. Once peaks are identified, CDP assigns them a unique label representing a cluster.

However, the association rule of CDP for the remaining points consists of inheriting the label of the closest point with a higher density, which ends in being a density peak. Although this generic rule is applicable to any cluster shape, cases remain where such a local criterion is not optimal (i.e., it associates a point disregarding how the other points are associated).

In this work, we propose to substitute the local association rule of CDP, with the solution of a global optimization problem on a graph, bringing two main contributions. First, a global vision on the dataset allows finding globally optimal solutions not identifiable with a local choice. Second, we evaluate path properties rather than point-to-point differences. This allows defining path-cost functions that can be generically meaningful (i.e., large gap penalization). In addition, by providing examples of valid and invalid paths, a path classifier can be trained. This is used for easily adopting and adapting the proposed method to specific tasks and datasets, by providing examples rather than tuning parameters, within a semisupervised approach for data analysis.

We evaluate the proposed method (both with a generic path-cost function and with a trainable path classifier) on synthetic datasets designed to challenge clustering algorithms (*2*). Then, we provide two applications for analyzing real biomedical data. The first is the segmentation of highly nonconvex immune cells in confocal microscopy (grouping pixels in space). The second is the identification of different types of arrhythmia in electrocardiographic (ECG) signals. Our method obtained clustering results not obtainable by DBSCAN nor CDP.

In conclusion, our method exploits network information (*6*) and the integration of possibly existing knowledge in line with a theory-guided data science paradigm (*7*). We consider it as a step toward machines (fast, operation based) capable of working collaboratively with humans (slow, practice based) (*1*).

## RESULTS

### Comparison with CDP on a simplified dataset

**Fig. 1:**
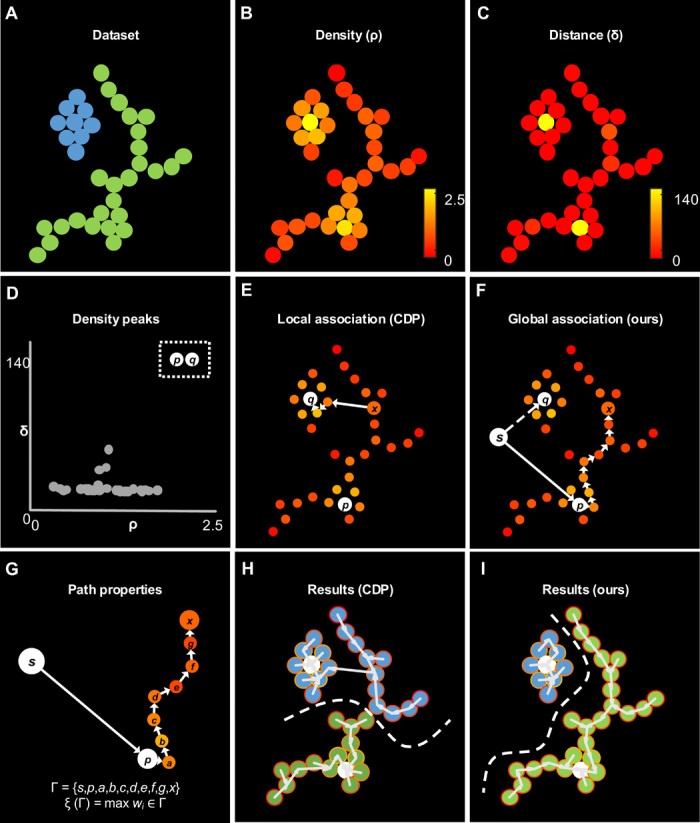
Association rules on a simplified example. (**A**) Raw data points in a 2D space. (**B**) Color-coded point density (ρ). (**C**) Color-coded distance to the closest point with a higher density (δ). (**D**) Density-distance plot for selecting density peaks (dashed box), characterized as outliers with sufficiently high density and distance. In this case, the density peaks correspond to the points *p*, *q* in (E) and (F). (**E**) Local association rule of CDP connecting a point *x*, to the closest point with higher density, until this is a density peak. This happens regardless of the other connections. (**F**) Global association rule of the proposed method, which computes the optimal path to connect a starting node *s* to a generic point *x*, passing by density peaks. (**G**) The path from *S*, via *p*, to *x* can be evaluated according to its properties, such as the maximum gap included. (**H**) Color-coded results of CDP. (**I**) Color-coded results of the proposed algorithm.

In CDP, a point is associated with the closest point with a higher density. This local rule takes place regardless of how the other points are associated, and there exist cases where artifacts are introduced. An example is illustrated in Fig. 1A, where a long-thin projection of a nonglobular cluster and a second globular-like cluster are in close proximity. The estimated point density (Fig. 1Β) and the distance to the closest point with a higher density (Fig. 1C) are used to find density peaks in a density-distance plot (Fig. 1D). For a generic point *x*, the association rule of CDP connects it to the wrong density peak *q* (Fig. 1D, white arrows). By contrast, our algorithm computes the path optimally connecting *x* to a density peak, which turns out to be *p* (Fig. 1F, white arrows). Although local heuristics would not consider that path (i.e., longer, nonsmooth density variation), our algorithm identifies it by solving a shortest path problem in which paths having multiple small gaps rather than fewer but larger gaps are preferred. This has been expressed using a minimax path-cost function (Fig. 1G). As a result, the thin portion of the cluster is correctly associated by our algorithm (Fig. 1, H and I). We consider the differences in this example relevant. Analyzing the cuts introduced by the two algorithms (Fig. 1, dashed lines), CDP introduces a visually nonrealistic cut, without the possibility of specifying how a cut should (or should not) be. By contrast, our algorithm obtained a visually realistic boundary by specifying desired path properties. The same result can be obtained using CDP to initially identify multiple smaller clusters (i.e., selecting more density peaks) and subsequently applying a graph-based algorithm to join them. However, this would require selection of density peaks that are not emerging to a smaller extent from the density-distance plot. Moreover, it would require specifying how to join subclusters at a higher level of abstraction (subgroup-to-subgroup similarity). Our algorithm solves this issue at the root and allows specifying how to group points at a lower level of abstraction, which is useful when pairwise-to-pathwise knowledge is available.

### Algorithm for global optimization

We consider clustering as the problem of associating each point to a density peak with the optimal set of paths. This problem is modeled as a single-source shortest path (SSSP) on a graph (Fig. 1F and fig. S1). A globally optimal solution is computed by Dijkstra’s SSSP algorithm (*8*), adopting a customized path-cost function.

To explain this in more detail, let us define the following quantities.

Let *G*(*V*, *E*, *w*) be a weighted graph where a node *v* ∈ *V* is connected to another node *u* ∈ *V*, *v* ≠ *u* through an edge *e*_*v*,*u*_ ∈ *E* with an associated cost *w*_*v*,*u*_ : *E* → ℝ_+_. *w*_*u*, *v*_ is a distance metric (i.e., Euclidean distance).

Let ρ*_v_* be the density of a given node *v* ∈ *V*, defined as the number of points in a weighted neighborhood. See (*5*) for an implementation where a Gaussian kernel is used to estimate ρ*_v_*.

Let δ*_v_* = min ∥ *v* − *u* ∥ ∀*u* ∈ *V* such that ρ*_u_* > ρ*_v_*, which is the distance of *v* to the closest point *u* with a higher density.

Let *P*_τ_ ⊂ *V*, *P*_τ_ = {*v*∣*v* ∈ *V*, ρ*_v_* > τ_ρ_ ∩ δ*_v_* > τ*_d_*}, where 0 < τ_ρ_ < max (ρ),0 < τ_δ_ < max (δ) are two arbitrary thresholds. We call the points in the set *P*_τ_ density peaks, i.e., nodes whose local density and distance to the closest point with a higher density are both sufficiently high.

Let *s* be a fictitious node added to *G* and connected to each density peak with an edge having a negligible cost ϵ (fig. S1).

The goal is to find the SSSP toward all the points with *s* as source, corresponding to the minimal spanning tree having *s* as root. Let Γ = {*s*, …, *x*} be the shortest path connecting the starting node *s* to an arbitrary point *x*. It is possible to show for reductio ad absurdum (proof S1) that *x* is reached, passing through a single density peak (and possibly intermediate points). In this case, *x* belongs to the cluster containing *p*.

Let *w*_*i*,*j*_ be the Euclidean distance between two nodes. If *G* is fully connected, the usage of a path-cost function that sums the cost of edges in the path does not penalize large gaps nor the problem of associating *x* to the closest but possibly not optimal peak *p*. Therefore, we describe the position of *x* with respect to a density peak by evaluating the path connecting them. Properties of the path, such as mediating elements (*9*), textures (*10*), density patterns, or user-defined metrics, can be used to associate a meaningful cost.

Dijkstra’s SSSP algorithm can be adopted to compute the shortest paths on graphs if the path cost is not decreasing when extending a path. Being ξ : G → R, ξ(Γ) = *c* the cost of a path Γ ∈ G, the following should be guaranteed

$$\xi (\Gamma =\{s,x_{i}\})\leq \xi (\Gamma \prime =\{s,x_{i},x_{i+1}\})$$ (1)

Several functions respect Eq. (1). Among these, cumulative functions sum the weights of edges included in the path $\xi_{p}(\Gamma )=\sum_{i,j\in \Gamma }{\left({|w_{i,j}|}^{p}\right)}^{\frac{1}{p}}$. Values of *p* ≥ 1 penalize edges with high cost, preferring (if possible) alternative multiple edges with lower cost. Generally, *p* > 1 acts a regularizer, preferring edges with similar value.

When *p* → + inf, the total cost of a path is given by the edge with maximum cost in the path. This metric is used in the minimax formulation, which defines for a generic path Λ the cost ξ_inf_(Λ) as max(*w*_*i*, *i*+1_), *i*, *i* + 1 ∈ Λ. Λ is “minimax” if its cost is minimum with respect to all the existing paths from the same source to the same destination

$$\Gamma \;\prime \prime \text{minimax}\prime \prime \;\text{if}\;\xi_{\text{inf}}(\Gamma )\leq \xi_{\text{inf}}(\Lambda )\;\forall \Lambda \neq \Gamma$$

#### Trainable path-cost function

The Dijkstra algorithm incrementally extends a set of shortest paths by evaluating the cost of the possible extensions. We exploited this behavior and used a custom function for computing the cost of an extended path, based on the last path fragment. Let Γ_1_ = {*s*, *x*_1_, *x*_2_, …, *x*_*n*−*w*_, …, *x_n_*} be a path from *s* to *x_n_* with associated cost *c*_1_. The cost of the extended path Γ_2_ = {*s*, *x*_1_, *x*_2_, …, *x*_*n*−*w*_, …, *x_n_*, *x*_*n*+1_} can be evaluated by means of a function that considers the cost until *x_n_* (corresponding to *c*_1_), and the path fragment *x*_*n*−*w*_, …, *x_n_*, *x*_*n*+1_, where *w* + 1 is the number of nodes in the fragment. In the original formulation of the Dijkstra algorithm, this is computed by adding to *c*_1_ the cost of the edge from *x_n_* to *x*_*n* + 1_. Instead, we propose to evaluate properties of a fragment including *w* nodes (more than one). Therefore, this fragment can be evaluated by a path classifier trained on a fixed number of elements.

Among the possible features to describe such a path fragment, we propose the density profile as the ordered density values of the nodes in the fragment. This opens the possibility to integrate existing knowledge in a supervised learning approach to prefer certain paths with respect to others by training a path classifier providing examples of admissible and nonadmissible paths.

### Reconstruction of heterogeneous synthetic shapes

**Fig. 2:**
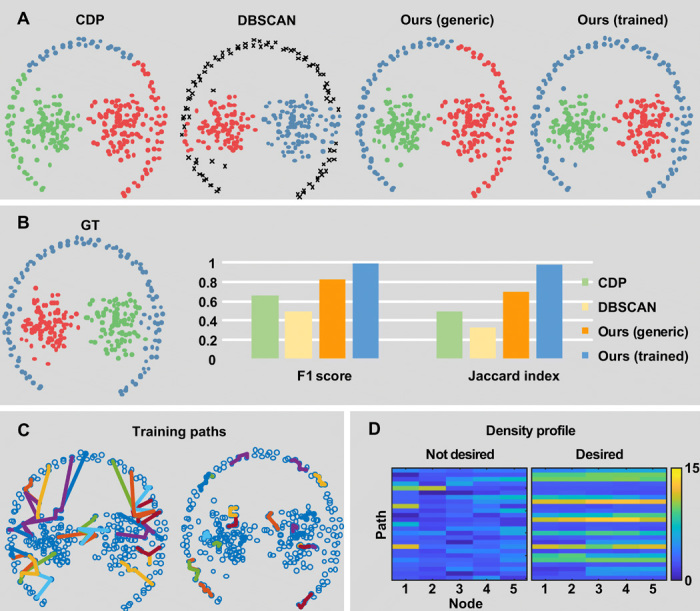
Evaluation on a synthetic dataset with heterogeneous structures. (**A** and **B**) The performances of the proposed method are evaluated on the synthetic dataset *01_chang_pathbased* provided in ClustEval (*2*). This includes groups with heterogeneous structures (i.e., a thin-elongated structure surrounding two globular-like structures). Our method, with a generic minimax path-cost function, partially improved the results produced by CDP and DBSCAN (A and B) with respect to the ground truth (GT). By contrast, by training a path classifier, the algorithm achieved full performances with an F1 score ≥0.99 and a Jaccard index ≥0.98. (**C**) Training paths composed of 25 examples of desired paths and 25 examples of undesired paths. (**D**) Path features corresponding to the density profile (vector including the density of the nodes in the path).

To benchmark the proposed algorithm, we benefit from the availability of the ClustEval platform (*2*), which provides synthetic datasets specifically designed to challenge clustering algorithms, together with an evaluation methodology. Among these datasets, we report the comparison with respect to other common methods on an example categorized as difficult, which visually contains heterogeneous structures (i.e., two globular-like clusters, surrounded by one thin-elongated cluster) (Fig. 2A). Our method achieves comparable performances to state-of-the-art algorithms when the generic minimax path-cost function is used (Fig. 2, A and B, orange columns).

However, our method outperforms the other algorithms when trained with examples of valid and invalid paths (Fig. 2, A and B, blue columns). In this example, 25 fragments of good paths and 25 fragments of bad paths composed by five points, respectively (Fig. 2C), were automatically generated from the ground truth and were described using the density of points as feature vector (Fig. 2D). In comparison to CDP, our method allowed correct clustering of the outermost elongated structure while still keeping separate the two central globular clusters. We provide two scripts to train the algorithm. In *demo_trainable.m* (data file S2), we provide the code for training the algorithm from an available (but not necessarily complete) ground truth. In *demo_interactive.m* instead, a GUI (graphical user interface) allows a user to visually inspect the dataset and annotate valid/invalid paths. A video showing an example of interactive training is provided in movie S1. Quantitative results on the remaining datasets included in ClustEval are provided in fig. S2A. Among these, in fig. S2B, we zoom in another challenging dataset where the trainable version of the algorithm allowed clustering of heterogeneous groups. A performance degradation assay with respect to the number of training paths and the length of the path fragments is provided in fig. S3.

#### Robustness to density peak detection

**Fig. 3:**
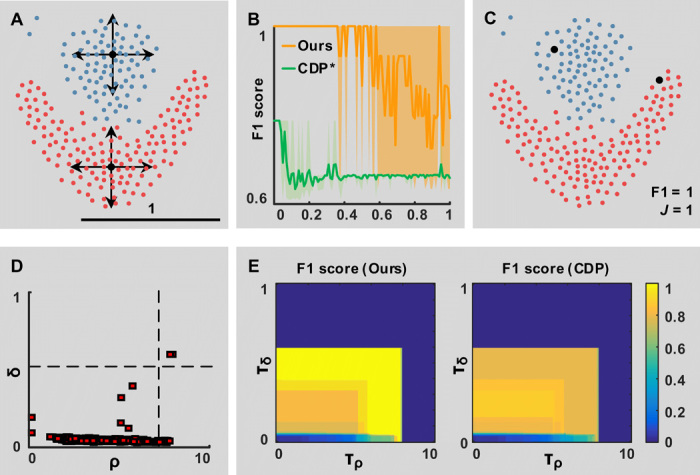
Robustness to density peak detection. (**A**) Color-coded ground truth including two clusters in close proximity (red and blue) from the synthetic dataset *04_fu_flame* provided in ClustEval. Points colored in black, at the intersection of the arrows, are the identified density peaks, which are used as seeds by the proposed algorithm. The position of seeds is perturbed by a displacement of max ±1 (black line). (**B**) Performance degradation with respect to the maximum perturbation. Results are obtained with *n* = 5 replicas with a random perturbation direction. CDP* refers to a modified version of CDP, forced to associate points to the perturbed seeds. (**C**) Color-coded results of the proposed method using the seeds colored in black. (**D** and **E**) Robustness to the setting of τ_ρ_ and τ_δ_ parameters. The optimal settings of the parameters (D) identifies two outliers of the δ − ρ graph. (E) Variations from the optimal settings include additional subclusters, lowering the performances of the algorithm.

Results of the proposed algorithm depend on the initial identification of density peaks. Figure 3 includes a performance assay, evaluating the F1 score of the proposed method with respect to an artificial perturbation on the position of density peaks (Fig. 3A). In the example *04_fu_flame* from ClustEval (*2*), the proposed method tolerates perturbations up to 30% of the dataset size without decrease in performance (Fig. 3B). Above this level, the algorithm exhibited a variable and generally decreasing behavior that was dependent on the position of the peaks with respect to the conformation of the dataset. More precisely, when two seeds were randomly assigned to points in the same cluster, an undesired partition was introduced, lowering the performances. Perturbations keeping seeds in two separated clusters are better tolerated (Fig. 3C).

The identification of density peaks depends on the setting of the threshold parameters τ_ρ_ and τ_δ_. These two parameters can be set to select only the outliers of a density-distance plot [see (*5*)] (Fig. 3D). Variations from the optimal values that do not select additional points as density peaks keep the maximum achievable performance. Variations that include additional points result in the identification of an increased number of clusters, lowering the performance with respect to the ground truth labels (Fig. 3E).

#### Noise handling

The proposed method can identify points that are suitable to be considered as noise by analyzing the path connecting them to a cluster. This can be achieved, for instance, by introducing a cutoff threshold on the path cost. When using a trainable path-cost function, examples of paths including noisy points can be used to penalize these connections. Automatic thresholding methods such as Otsu thresholding (*11*) can be used to set the cutoff distance. Considering that a density profile can be used as path descriptor, this strategy is in line with the criterion of DBSCAN, which considers as noise those points not density reachable from a cluster.

**Fig. 4:**
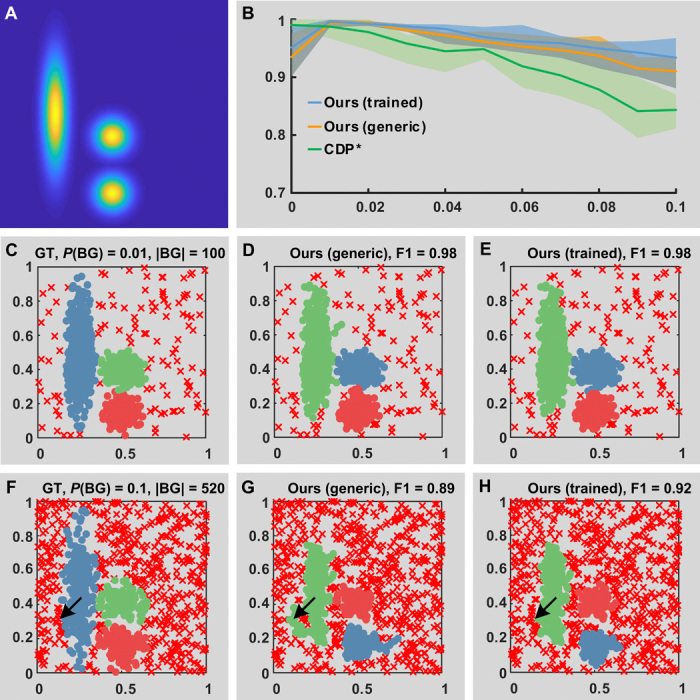
Robustness to background noise. (**A**) Synthetic probability distribution from which point distributions are drawn. Lighter areas have the maximum probability. The area in blue is a background with uniform probability from 0 to 0.1, corresponding to a number of points from 10 to 52% of the total. (**B**) Quantitative performance degradation assay with respect to the value of background probability. A total of 1000 points are drawn from the probability distribution in (A), varying the background uniform probability from 0 to 0.1. For each level of background probability, *n* = 5 replicates are generated. Bold lines refer to the mean of the F1 score among the replicates, while the shaded lines correspond to the range. Three peaks were selected using γ ranking (*5*). (**C** to **E**) Qualitative results with a background probability of 0.01, corresponding to 900 points from the clusters and 100 points in the background. Cluster labels are color coded. Red x correspond to noise. (**F** to **H**) Qualitative results with a background probability of 0.1, corresponding to 480 points from the true clusters and 520 points in the background. Arrows indicate an aggregation of points in the background. The trained algorithm detects those points as noise.

A benchmark of the proposed method in the presence of background noise is reported in Fig. 4. To this end, we generated a synthetic probability distribution with three density peaks and a uniform background with a different probability (Fig. 4A). The proposed method exhibited average better results than CDP, with a decreased slope of the F1 score with respect to the background noise level (Fig. 4B). With a low level of background noise, both a minimax path-cost function and a trained path-cost function achieved similar performances (Fig. 4, C to E). For higher levels of noise, a path-cost function trained to associate high cost to paths including noise (Fig. 4, F to H) yielded an improved recognition of background.

### Application on biomedical data

#### Segmentation of immune cells in confocal microscopy

Microscopy images are considered challenging spatiotemporal biomedical datasets (*12*, *13*). This is particularly relevant for images of immune cells, which exhibit high plasticity, both globular and nonglobular shapes, possibly in contact (*14*, *15*). Considering segmentation as a grouping task, the aforementioned properties represent challenges for clustering algorithms (i.e., heterogeneous shapes in close proximity). Moreover, they remain challenging for state-of-the-art bioimaging software, which requires manual correction of results, lowers usability (*16*), and compromises reproducibility.

**Fig. 5:**
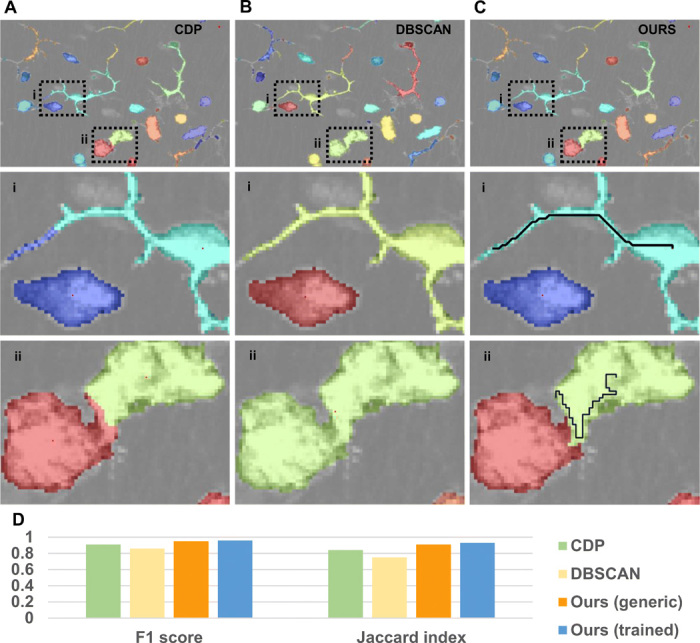
Application for segmentation of immune cells. (**A** to **C**) Qualitative results (color-coded labels) produced by different density-based clustering algorithms for clustering pixels in space. Images show a confocal microscopy acquisition of murine immune cells, labeled with CD11c + GFP. (i) Magnification showing a cell with dendritic morphology close to a cell with globular shape. (ii) Magnification showing two cells with globular shape in contact. CDP (A) using a Euclidean metric correctly separates touching cells but associates a piece of dendrite to the wrong cell. DBSCAN (B) correctly reconstructs the shape of dendritic cells but is not able to separate touching cells with the same density-reachability criterion. The proposed method (C) correctly associates the dendrite of the dendritic cell and separates the touching cells. Black lines indicate the optimal path followed by the algorithm, from the cell centroid (density peak) to a point in the dendrite and in the touching region, respectively. (**D**) Quantitative performances. The F1 score and the Jaccard index are computed with respect to the ground truth. Here, the trained version of the algorithm achieved similar performances to the generic version. These are bounded because of the separation of cells with increased size into multiple subclusters.

Here, we apply our clustering algorithm for grouping pixels. We assume that other image-processing tasks such as background removal have been already executed with specific software such as Ilastik (*17*), Trainable Weka Segmentation (*18*), or Imaris (Bitplane). Qualitative results are presented in Fig. 5. Similar to the example in Fig. 1, in Fig. 5A, the local association rule of CDP yields to the wrong association of a thin dendrite (i) but correctly separates touching cells (ii). In Fig. 5B, DBSCAN correctly reconstructs the shape of dendritic cells but does not separate touching cells. In Fig. 5C, our algorithm, penalizing large gaps in the paths, connects the pixels to the correct density peaks in both the cases.

In this example, training the algorithm does not increase performances substantially. These are limited because more than necessary density peaks are identified as starting points. As a consequence, some cells are split into multiple parts. To merge these subclusters, a different metric can be used for computing the δ measure for each peak. Alternatively, methods for merging multiple clusters such as (*19*) can be applied for postprocessing.

#### Identification of different heart rhythms

**Fig. 6:**
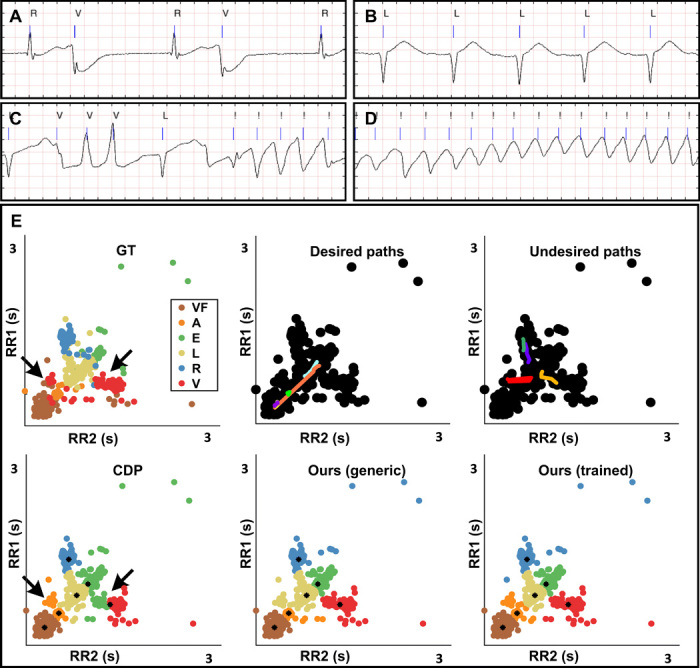
Recognition of different heart rhythms. (**A** to **D**) Fragments of an ECG signal from a patient with several types of arrhythmia. Blue lines indicate a beat (systole). Cardiologists labeled six different types of beats “A, V, E, R, L, and !,” associated with different rhythms. Atrial/ventricular/ectopic beats “A/V/E” are extrasystoles close to the previous beat that can induce a pause in the rhythm (A). Right bundle branch block “R”: beat appearing in this patient between two V. Left bundle branch block “L” (B): normal beat type for this patient (similar RR1 and RR2 varying linearly in tachycardia and bradycardia). Runs of ventricular extrasystoles not followed by long pause are also reported (C). Ventricular flutter “!,” later indicated as “VF,” a critical condition characterized by extremely fast contractions (D). (**E**) Heartbeats represented as points, described according to their distance from the previous and to the next beat (RR1 and RR2). Our method recognized ventricular extrasystoles (red), which are otherwise mixed with L beats by CDP. The trained version of the algorithm further allowed identification of a group composed of mixed atrial and ventricular extrasystoles (orange).

Long-term ECG records are widely used to capture the dynamic behavior of the heart, which may change over time and exhibit different rhythms. Here, we applied clustering analysis to automatically identify different rhythms in a complex ECG record showing various types of arrhythmia (*20*). Arrhythmia affects the distance between heartbeats (Fig. 6A). To this end, we described each heartbeat with two parameters corresponding to the distance (in seconds) from the previous (RR1) and the next (RR2) heartbeat. These two parameters are similar in sinus rhythms (Fig. 6B) and vary linearly according to the heart rate. However, in the presence of extrasystoles, these may change. Extrasystoles are beats that stay closer to the previous beat and are possibly followed by a long pause. There are two main types of extrasystoles: atrial and ventricular. The second has an increased alteration of the distances. Life-threatening arrhythmias such as ventricular tachycardia and flutter result in extremely short distances between beats (Fig. 6, C and D), compromising the pump function of the heart. In results presented in Fig. 6E, our algorithm identified six different rhythms.

Similar to the ground truth (manually annotated by a group of cardiologists), our algorithm identified groups corresponding to ventricular flutter (brown), isolated ventricular extrasystoles (red), isolated atrial extrasystoles (orange), and right bundle branch block (blue). By contrast, the algorithm identified two different subtypes (instead of one) of sinus beats, one associated with lower heart rate (gold), and one associated with higher heart rate (green).

With respect to CDP, our algorithm removed an unrealistic boundary that was cutting a piece of the ventricular extrasystole cluster (red). In CDP, these beats were joined by mistake to the normal beats (green), while in our algorithm, they are correctly joined with the ventricular extrasystole group (red). The trained version further allowed a group of mixed atrial-ventricular extrasystoles to be found, which were associated by mistake to the normal beats by the untrained version.

## DISCUSSION

The proposed method inherits the advantages of density-based clustering methods but, using a global association rule, is able to overcome the limitations of local connectivity rules. This is in line with the work by Tenenbaum *et al.* (*21*), which uses global optimization (i.e., shortest path computation) to represent the global structure of a dataset in a lower dimensional coordinate system. Clustering can be interpreted as a special case of dimensionality reduction, where points are represented by a single discrete coordinate corresponding to the assigned label.

The application of the proposed method requires the initial detection of density peaks as described in (*5*). Although this step may hamper the application to datasets where density peaks do not exist, CDP demonstrated broad applicability to challenging datasets (*2*) where density peaks become easily identifiable as outliers, with high density and high distance, in a density-distance plot. Outliers of the density-distance plot can be selected as points above two thresholds, τ_ρ_ and τ_δ_. These thresholds can be set either manually by inspecting the density-distance plot, or automatically [i.e., via Otsu thresholding (*11*)], or to meaningful values for applications where the expected cluster size and density are known. Alternatively, points can be ranked according to a score and the top *N* points selected as density peaks (*N* = number of desired clusters). In addition, a combined approach based on thresholding and ranking can be adopted.

There might be cases in which density peaks are not obvious to identify. This can happen in the presence of clusters composed of multiple subclusters. Peaks of these subclusters may arise in the density-distance plot. In this case, a hierarchical approach is suggested.

Recently, Du *et al.* (*22*) proposed an approach that facilitates the identification of density peaks using the graph distance to compute δ. Two points in the same cluster are more likely to be connected by a path with low cost. Density peaks that are in distinct clusters instead are more likely to be connected by a path with high cost. Hence, the graph distance of density peaks is likely to be higher than the graph distance of arbitrary nonpeak points in the same cluster, resulting in higher values of δ for density peaks.

The proposed method generalizes to high-dimensional datasets if they can be represented as a graph and a point density can be estimated. Common methods to estimate point density rely on Euclidean distance to define a neighborhood. However, Euclidean distance loses significance with an increasing number of variables (*23*). Hence, it should be computed only in a sufficiently restricted neighborhood. Figure S4 reports a benchmark on synthetically generated datasets, including multidimensional distributions using Euclidean distance as metric to build the graph. The proposed algorithm exhibits comparable performances to CDP.

Arbitrary nonnegative metrics for edge cost are supported by the proposed methods. Figure S5 reports an application on real high-dimensional data from the bone marrow leukemia dataset (*2*, *24*). Such a dataset includes the gene expression level of 999 genes from 38 samples of patients with three different types of leukemia. The proposed method correctly identified three groups with comparable performances to CDP. In this case, the graph was built with an edge cost derived from the Spearman correlation.

Alternatively, graph pruning can be used together with a cumulative Euclidean path-cost function to limit the connectivity of each node within a restricted neighborhood. Methods for dimensionality reduction can further be used for data preprocessing. Among these, global and nonlinear approaches, such as Isomap (*21*), are preferable with respect to principal components analysis for highly nonconvex datasets.

The proposed path-based formulation supports the expression of a path cost by evaluating path properties. A nondecreasing path-cost function allows solving the proposed clustering problem using Dijkstra’s SSSP algorithm. Among the possible path-cost functions, minimax penalizes paths with large gaps, which is a generically desired property. Other nonlinear functions can be designed for specific tasks.

Dijkstra’s SSSP algorithm exhibits a lower than quadratic computational complexity that can be reduced for sparse graphs. Moreover, it does not require storage of the adjacency matrix on memory and can be speeded up by adopting heuristics for optimal path-finding or graph-partitioning techniques. Another advantage of the Dijkstra algorithm is that it supports graphs with multiple edges between two nodes. This opens the possibility to express the distance between two nodes using multiple edges and metrics. During the optimization process, the edge and the metric that are more convenient for the solution will be automatically selected. Therefore, we consider this method suitable to be adapted and optimized for specific applications.

Last, existing knowledge can be integrated by defining an appropriate path-cost function, which can be obtained, for instance, by training a path classifier with examples provided by humans or available ground truths. Therefore, it represents an intermediate method between machine and human computation for tasks that would benefit from a collaborative effort (*1*, *25*).

## MATERIAL AND METHODS

### Data generation

#### Confocal microscopy

For data shown in Fig. 5, immunofluorescence confocal microscopy was performed using a Leica TCS SP5 microscope, with sequential acquisition to limit signal cross-talking. A murine trachea was collected and fixed in 4% paraformaldehyde at 4°C for 1 hour. Tracheas were cut in two halves along the long axis. Pieces were placed on a microscopy slide and embedded in Fluoromount Aqueous Mounting Medium (Sigma-Aldrich). Three-dimensional (3D) images were acquired using an HCX PL APO CS 20×/0.7 oil immersion objective, and *z*-step was 1 μm for a total depth ranging from 50 to 112 μm. The bioimaging software Bitplane Imaris (v 9.2.1) was used for computing the 2D maximum intensity projections and for manual cell segmentation. The same software (Coloc functionality) was used to exclude the background and to create a channel containing only the points belonging to Cd11c-GFP^+^ cells. This dataset along with the ground truth is provided in data file S1 using the same format described in (*2*).

Mice were maintained in specific pathogen–free facilities at the Institute for Research in Biomedicine, Bellinzona. Experiments were performed in accordance with the Swiss Federal Veterinary Office guidelines, and animal protocols were approved by the veterinarian local authorities.

#### Synthetic datasets

The dataset presented in Fig. 1 is provided in data file S1. The synthetic point distributions in Fig. 4 were generated by the script *benchmark_noise.m* in data file S2. More precisely, three bivariate distributions were overimposed to a uniform background. This was achieved by creating a probability matrix *M* of size 200 × 200. Then, 1000 samples were extracted from the matrix as follows. For each sample, a pair of random coordinates (*x*, *y*) and a random number *p*, from 0 to 1 with uniform probability, were generated. If *M*(*x*, *y*) > *p* and the point was not already selected, then it was included into the dataset.

Points in the background were selected if the probability of the background was higher than the randomly generated number. Controlling the probability of the background allowed selection of a different number of points in the background with respect to the points in the other distributions.

The synthetic high-dimensional datasets in fig. S4 were generated using MDCGen (Multidimensional Dataset Generator for Clustering) (*26*), configured to generate two multivariate Gaussian distributions. The number of dimensions ranged from 2 to 100. For each dimension, 15 different distributions (mean and variance) were randomly computed. Code is provided in the script *benchmark_dimensions.m* (data file S2).

### Implementation

The proposed algorithm was implemented in MATLAB r2017b. A demo for both the generic (minimax) and trainable algorithms are provided respectively in *demo_generic.m* and *demo_trainable.m* in (data file S2). Moreover, an interactive example where the user can visually draw examples of valid paths and invalid paths on a 2D dataset is provided in *demo_interactive.m* and in movie S1.

Dijkstra’s SSSP algorithm, which is at the core of the proposed method, was implemented using vectorization and outperforming the *graphshortestpath()* routine distributed with the Bioinformatics Toolbox of MATLAB r2017b.

The following libraries have been used:

• DensityClust.m—implementation of the CDP clustering algorithm (fileexchange/53922-densityclust)

• dbscan.m—implementation of the DBSCAN clustering algorithm (www.peterkovesi.com/matlabfns/Misc/dbscan.m)

• FitCSVM—implementation of a supported vector machine classifier (MATLAB Bioinformatics Toolbox)

• mdcgen-matlab (*26*)—generator of high-dimensional datasets for clustering (https://github.com/CN-TU/mdcgen-matlab).

### Path classifier

In the example provided in Fig. 2 (C to E), a support vector machine with radial basis function kernel was trained on 25 desired paths and 25 nondesired path fragments. Results can be reproduced, and training data can be generated using the MATLAB script *demo_trainable.m* provided in data file S2.

### Evaluation

For the quantitative benchmarking of the proposed method with respect to the ground truth, the F1 score and the Jaccard index (*J*) were computed according to (*2*). These were defined as follows: *F*1 = 2 * (Recall * Precision)/(Recall + Precision). *J* = TP/(TP + FN + FP), where TP is the true positive, FN is the false negative, FP is the false positive, and TN is the true negative. Recall = (TP)(TP + FN), Precision = TP/(TP + FP).

## Supplementary Material

aax3770_Data_file_S2.zip

aax3770_Movie_S1.mp4

aax3770_SM.pdf

aax3770_Data_file_S1.zip

## SUPPLEMENTARY MATERIALS

Supplementary material for this article is available at http://advances.sciencemag.org/cgi/content/full/5/10/eaax3770/DC1 Proof S1. Unique cluster assignation. Fig. S1. Graph structure. Fig. S2. Results on the 12 synthetic datasets provided by ClustEval. Fig. S3. Performance degradation with respect to number of training paths. Fig. S4. Benchmark on high-dimensional synthetic datasets. Fig. S5. Results on the bone marrow leukemia dataset. Data file S1. Additional datasets used in this article. Data file S2. MATLAB source code of both the generic and trainable algorithm. Movie S1. Demo showing the training procedure.

## Appendix

View/request a protocol for this paper from *Bio-protocol*.
