# Supplementary material for: A trainable clustering algorithm based on shortest paths from density peaks
Source: Sci Adv. 2019 Oct 30;5(10):eaax3770. doi: 10.1126/sciadv.aax3770 (PMC7051829; doi:10.1126/sciadv.aax3770)
Supplement: aax3770_SM.pdf [file aax3770_SM.pdf]

Science Advances
AAAS

advances.sciencemag.org/cgi/content/full/5/10/eaax3770/DC1

Supplementary Materials for

A trainable clustering algorithm based on shortest paths from density peaks

Diego Ulisse Pizzagalli, Santiago Fernandez Gonzalez*, Rolf Krause*

*Corresponding author. Email: santiago.gonzalez@irb.usi.ch (S.F.G.); rolf.krause@usi.ch (R.K.)

Published 30 October 2019, *Sci. Adv.* **5**, eaax3770 (2019)
DOI: 10.1126/sciadv.aax3770

The PDF file includes:

Proof S1. Unique cluster assignation.
Fig. S1. Graph structure.
Fig. S2. Results on the 12 synthetic datasets provided by ClustEval.
Fig. S3. Performance degradation with respect to number of training paths.
Fig. S4. Benchmark on high-dimensional synthetic datasets.
Fig. S5. Results on the bone marrow leukemia dataset.

Other Supplementary Material for this manuscript includes the following:

(available at advances.sciencemag.org/cgi/content/full/5/10/eaax3770/DC1)

Data file S1 (.zip format). Additional datasets used in this article.
Data file S2 (.zip format). MATLAB source code of both the generic and trainable algorithm.
Movie S1 (.mp4 format). Demo showing the training procedure.

Proof S1. Unique cluster assignation.

We want to demonstrate that only a single density peak is included in the shortest path from the dummy node s to a generic point x. Let $\mathbb{G}$ be the set of all the possible paths on the graph provided in (fig. S1), where a starting node s is connected only to a set of nodes P, namely "density peaks", with a negligible cost $\epsilon \in \mathbb{R}_+, \epsilon \to 0$. Now, let us assume the existence of a shortest-path $\Gamma = \{s, P_1, a, P_2, b, x\}$ from s to x such that two density peaks P_1 and P_2 are included. We encounter a contradiction since the cost of the sub-path $\{s, P_1, a, P_2\}$ is always greater than the cost of directly connecting $\{s, P_2\}$. Therefore, Γ is not a shortest path.

This proof is valid under the assumption that the cost of a generic path $\Gamma \in \mathbb{G}$ is evaluated by means of a non-decreasing function $\xi : \mathbb{G} \to \mathbb{R}, c = \xi(\Gamma), \quad \xi(\Gamma_1) \leq \xi(\{\Gamma_1, \Gamma_2\}) \quad \forall \Gamma_2 \in \mathbb{G}$.

Supplementary figures

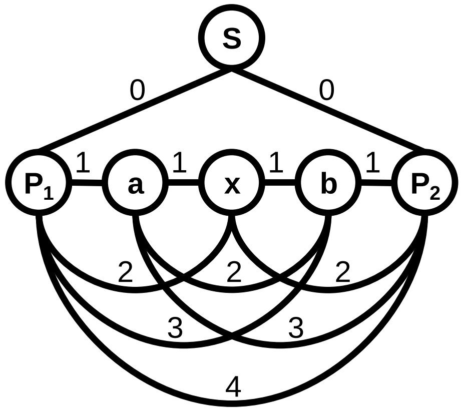

Fig. S1. Graph structure. Each density peak P_i is connected to a dummy node s with a negligible cost ϵ. All the remaining points $\{a, b, ...\}$ are connected on a graph that guarantees their reachability from s. Despite in this example we provide a fully connected graph, the proposed algorithm supports sparse/pruned graphs guaranteeing this condition. Additionally, the algorithm supports non-negative edge costs which can be derived from an arbitrary point-to-point distance (i.e. Euclidean distance).

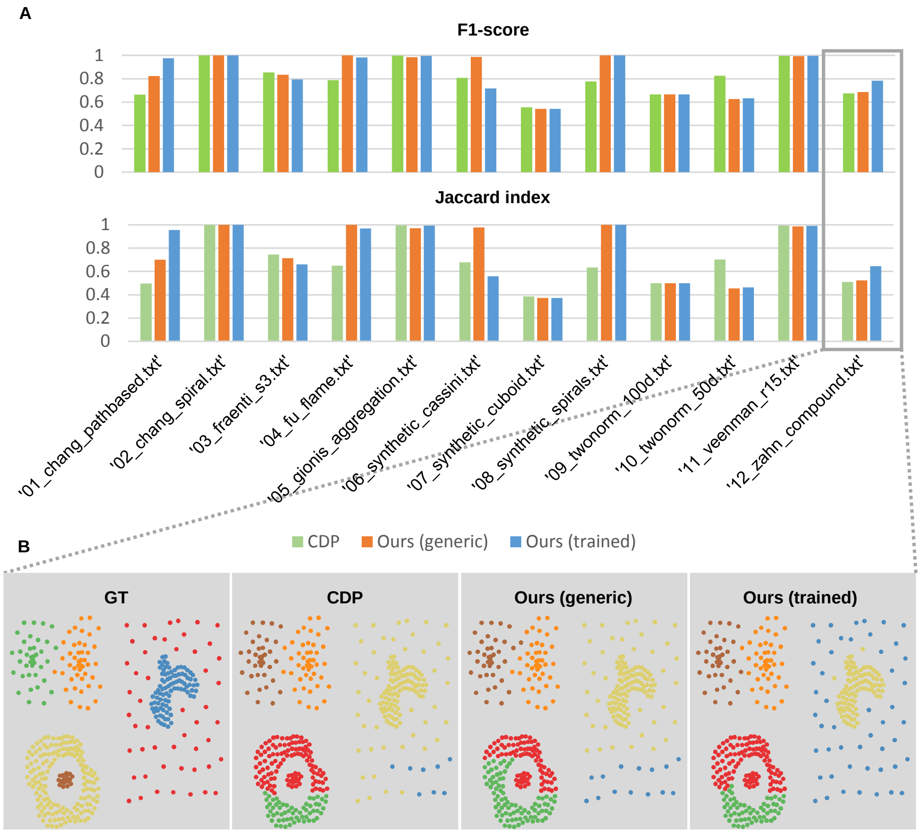

Fig. S2. Results on the 12 synthetic datasets provided by ClustEval. A. Quantitative results. **B.** Qualitative results on zahn_compound dataset including heterogeneous shapes.

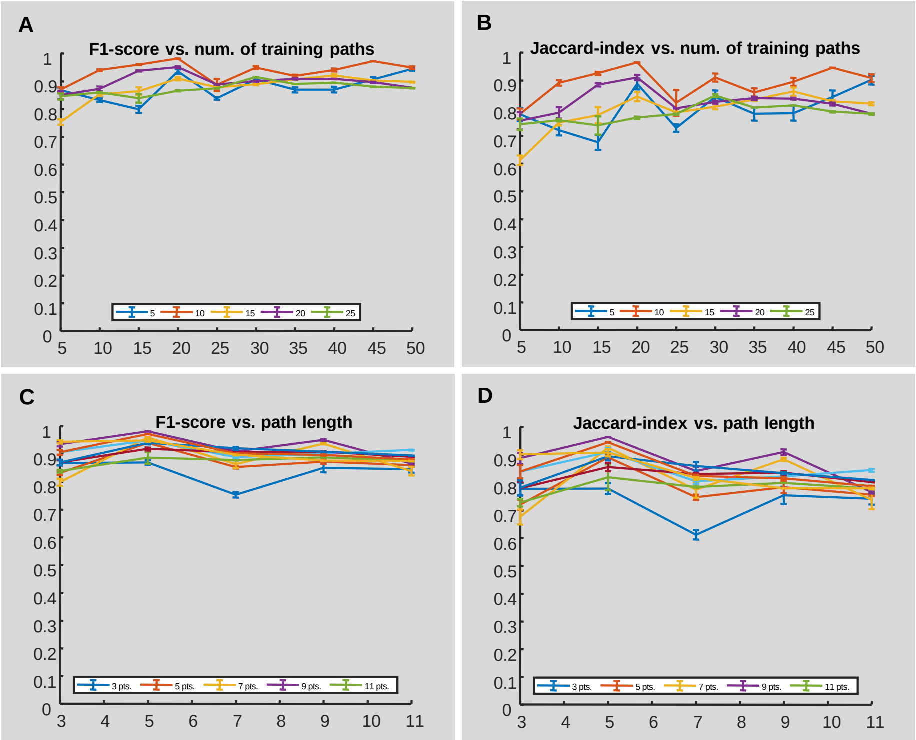

Fig. S3. Performance degradation with respect to number of training paths (A-B) and to the number of points in each fragment (path length) **(C-D)** on the dataset *01_chang_pathbased*. Mean and standard deviations using 5 different runs of the algorithms with the same parameters, but with random paths are provided.

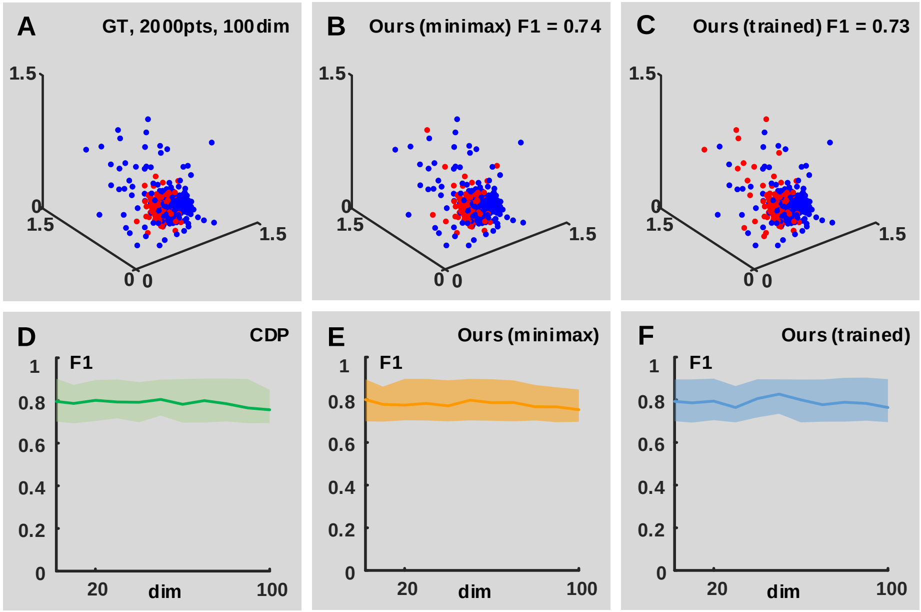

Fig. S4. Benchmark on high-dimensional synthetic datasets (A-C) Results on a synthetic generated dataset of 2000 points and 100 dimensions with two clusters. Three dimensions are shown. **(D-F)** F1-score varying the dimensionality. For each dimension, 15 datasets with two clusters of different size and position were generated. Bold lines refer to the mean F1-score, the shaded areas the range of CDP (D), proposed method using a generic minimax path cost function (E), and the proposed method trained with random paths (F).

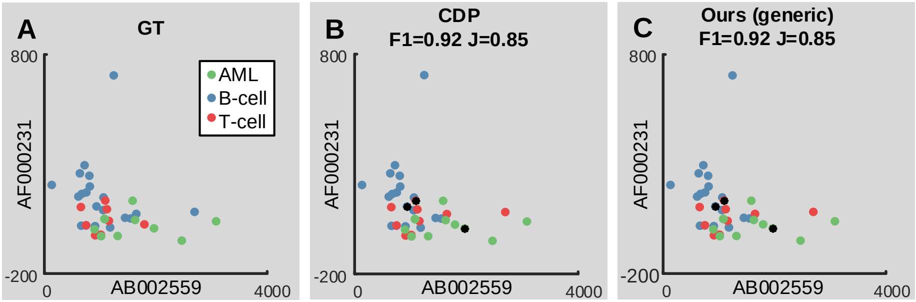

Fig. S5. Results on the bone marrow leukemia dataset (A-C) The proposed method was evaluated on a high dimensional dataset containing the expression of 999 genes on 38 samples from patients with three three different types of leukemia (AML - Acute Myeloid Leukemia, B and T cell leukemia). **A** Representation of two dimensions out of 999 with color coded points according with the Ground Truth (GT) provided in [2, 25]. (B,C) Quantitative and qualitative results of CDP and the proposed methods using a minimax path cost function. In this example and edge-cost derived from the Spearman correlation is used.
